# Toxicity of cannabidiol and its metabolites in TM3 mouse Leydig cells: a comparison with primary human Leydig cells

**DOI:** 10.1007/s00204-024-03754-x

**Published:** 2024-04-17

**Authors:** Yuxi Li, Qiangen Wu, Xilin Li, Patrick Cournoyer, Supratim Choudhuri, Lei Guo, Si Chen

**Affiliations:** 1 Division of Biochemical Toxicology, National Center for Toxicological Research, U.S. Food and Drug Administration, 3900 NCTR Road, Jefferson, AR 72079 USA; 2 Division of Genetic and Molecular Toxicology, National Center for Toxicological Research, U.S. Food and Drug Administration, 3900 NCTR Road, Jefferson, AR 72079 USA; 3 Office of the Commissioner, U.S. Food and Drug Administration, Silver Spring, MD 20993 USA; 4 Office of Food Additive Safety, Center for Food Safety and Applied Nutrition, U.S. Food and Drug Administration, College Park, MD 20740 USA

**Keywords:** Cannabidiol, 7-Carboxy-CBD, 7-Hydroxy-CBD, Male reproductive toxicity, mRNA-sequencing, TM3 mouse Leydig cells, Primary human Leydig cells, Apoptosis

## Abstract

Cannabidiol (CBD), one of the major components extracted from the plant *Cannabis sativa L.*, has been used as a prescription drug to treat seizures in many countries. CBD-induced male reproductive toxicity has been reported in animal models; however, the underlying mechanisms remain unclear. We previously reported that CBD induced apoptosis in primary human Leydig cells, which constitute the primary steroidogenic cell population in the testicular interstitium. In this study, we investigated the effects of CBD and its metabolites on TM3 mouse Leydig cells. CBD, at concentrations below 30 µM, reduced cell viability, induced G1 cell cycle arrest, and inhibited DNA synthesis. CBD induced apoptosis after exposure to high concentrations (≥ 50 µM) for 24 h or a low concentration (20 µM) for 6 days. 7-Hydroxy-CBD and 7-carboxy-CBD, the main CBD metabolites of CBD, exhibited the similar toxic effects as CBD. In addition, we conducted a time-course mRNA-sequencing analysis in both primary human Leydig cells and TM3 mouse Leydig cells to understand and compare the mechanisms underlying CBD-induced cytotoxicity. mRNA-sequencing analysis of CBD-treated human and mouse Leydig cells over a 5-day time-course indicated similar responses in both cell types. Mitochondria and lysosome dysfunction, oxidative stress, and autophagy were the major enriched pathways in both cell types. Taken together, these findings demonstrate comparable toxic effects and underlying mechanisms in CBD-treated mouse and primary human Leydig cells.

## Introduction

Cannabidiol (CBD), one of the main cannabinoids extracted from the cannabis plant *Cannabis sativa L.*, was first isolated in 1940s and its chemical structure was subsequently determined in 1960s (Huestis et al. 2019; Ujvary and Hanus 2016). In 2018, Epidiolex®, an oral CBD solution, was approved by the U.S. Food and Drug Administration (FDA) to treat seizures in patients (≥ 2 years of age) with Lennox–Gastaut syndrome and Dravet syndrome (U.S. FDA 2018). In 2020, the FDA approved Epidiolex oral solution for the treatment of seizures in patients (≥ 1 year of age) with tuberous sclerosis complex (U.S. FDA 2020). CBD became popular in recent years due to its purported health benefits and increased availability. Given the increasing public interest in CBD, it is essential to investigate potential adverse effects associated with its use. One potential concern is the reproductive and developmental toxicity of CBD, which has not been evaluated clinically, especially for long-term exposure effects (U.S. FDA 2019). Since 7-hydroxy-CBD and 7-carboxy-CBD are the two major circulating metabolites found in humans and experimental animals, and data remain scarce on their toxicity profile, it is also essential to evaluate the potential toxicities associated with 7-carboxy-CBD and 7-hydroxy-CBD (Kicman and Toczek 2020).

CBD-induced male reproductive toxicity has been reported in several animal models. For example, oral administration of CBD (30, 100, or 300 mg/kg/day for 90 days) to rhesus monkeys resulted in decreased testicular size and inhibited spermatogenesis as evidenced by smaller seminiferous tubules, fewer germ cells per tubule, and a lower mitotic index (Rosenkrantz et al. 1981). Rosenkrantz and Esber also observed a 30% decrease in testosterone at 300 mg/kg in rhesus monkeys after 90 days of treatment (Rosenkrantz and Esber 1980). Inhalation of CBD at doses of 0.6, 0.8, or 1.2 mg/kg in rats caused degeneration of the seminiferous tubules (the sperm factory) and interference with sperm maturation (Rosenkrantz and Hayden 1979). Intraperitoneally administered CBD to rats at a dose of 10 mg/kg decreased testosterone metabolism (Narimatsu et al. 1990). Delayed sexual maturation and small testes and fertility were observed in the offspring of rats after oral administration of CBD (75, 150, or 250 mg/kg/day) during both pregnancy and lactation (Huestis et al. 2019). Additionally, 21-day-old male Swiss mice treated orally 15 or 30 mg/kg CBD for 34 days and allowed to recover for 35 days exhibited reduced spermatozoa in the epididymis tail, decreased Sertoli cells at meiotic stage XII, and increased head abnormalities of sperm (Carvalho et al. 2018). Interestingly, 30 mg kg/day oral CBD exposure caused a 76% decrease in testosterone, although it still remained within the physiologically normal range (240–1100 ng/dL) (Carvalho et al. 2018). These studies imply that the administration of CBD is associated with male reproductive toxicity in animals; however, the mechanisms underlying CBD-induced reproductive toxicity are still unclear.

The decreased testosterone levels induced by in vivo CBD exposure prompted us to examine the effects of CBD on Leydig cells, which are mainly responsible for testosterone secretion in the testes. In a previous study, we examined the effects of CBD and its metabolites on primary human Leydig cells and observed that CBD caused G1 cell cycle arrest, decreased DNA synthesis, and induced apoptosis (Li et al. 2023a, b). In addition, both 7-hydroxy-CBD and 7-carboxy-CBD induced apoptosis in primary human Leydig cells. In the present work, we investigated the response of TM3 mouse Leydig cells to CBD treatment and its underlying mechanisms. TM3 Leydig cells are a proliferating mouse Leydig cell line that was originally isolated from immature mice (Matfier 1980). They have been widely used in mechanistic studies to investigate chemical-induced reproductive toxicity (Komatsu et al. 2008; Wang et al. 2019). In addition, we compared the transcriptomic changes induced by CBD in primary human Leydig and TM3 mouse Leydig cells. The results provide insights into the effects of CBD and its metabolites on Leydig cells across species and the potential underlying molecular mechanisms.

## Materials and methods

### Test chemicals and cell culture

CBD (Batch # NQSS1951, stated purity 100%), 7-hydroxy-CBD (Batch # BDG11596, stated purity 97.6%), and 7-carboxy-CBD (Batch # BDG11603, stated purity 97.4%) were purchased from Purisys (Athens, GA). The chemicals were dissolved in DMSO (Catalog # D8418, MilliporeSigma, St. Louis, MO) and stored as 100 mM solutions at − 20 °C.

The TM3 mouse Leydig cell line (ATCC® CRL-1714™) was purchased from ATCC (Manassas, VA). DMEM: F-12 Medium (Catalog # 30–2006™, ATCC) media-containing 5% horse serum (Catalog # 16050122, Gibco, Gaithersburg, MD), 2.5% fetal bovine serum (FBS, Catalog # S11150, R&D systems, Minneapolis, MN), 100 U/mL of penicillin, and 100 µg/mL of streptomycin (Catalog # 15140163, Gibco) were used to culture TM3 cells. TM3 cells were split every 2–3 days upon reaching 95% confluency and were maintained for up to 10 passages. Methods of culturing primary human Leydig cells were described in detail previously (Li et al. 2023a). Both types of cells were cultured at 37 °C in a humidified atmosphere with 5% CO_2_.

### Measurement of cellular viability

TM3 cells were seeded at a density of 1.7 × 10^5^ cells/well in 6-well plates for 24 h prior to treatment with 10–55 µM CBD, 10–55 µM 7-hydroxy-CBD, or 40–200 µM 7-carboxy-CBD for 24 h. Cells were then collected by trypsinization and centrifugation. The number of viable cells was determined using trypan blue (Catalog # T10282, ThermoFisher Scientific, Waltham, MA) on a Countess 3 automated cell counter (Catalog # AMQAX2000, ThermoFisher Scientific). Cell viability was calculated by normalizing the cell numbers of chemicals-treated groups to those of the DMSO controls. The concentration of chemicals that caused a 50% decrease of cell viability was estimated by Bayesian benchmark dose (BMD) methodology as BMD_50_ (Shao and Shapiro 2018).

### Lactate dehydrogenase (LDH) assay

The release of LDH into the cell culture medium was measured as previously described (Chen et al. 2018) to determine the extent of cell damage. Briefly, TM3 cells were seeded at a density of 5000 cells/well in 96-well plates for 24 h prior to treatment with CBD, 7-hydroxy-CBD, or 7-carboxy-CBD at designated concentrations for 24 h. After treatment, supernatants and cell lysates obtained by Triton™ X-100 treatment were collected from each well and mixed with reaction buffer containing 81.3 mM Tris, 203.3 mM NaCl, 0.2 mM NADH, and 1.7 mM monosodium pyruvate (pH 7.2). The absorbance at 340 nm was immediately measured four times at 1-min intervals using Cytation 5 plate reader (Agilent BioTek, Santa Clara, CA). The LDH release was calculated as follows: 100 × (OD 340 nm from Read #1—OD 340 nm from Read #4 of supernatant /OD 340 nm from Read #1—OD 340 from Read #4 of cell lysate).

### F-actin immunofluorescence staining

Immunofluorescence staining against F-actin was performed on TM3 cells that were cultured in µ-Slide18 Well chamber slides (Catalog # 81816, ibidi, Martinsried, Planegg, Germany). TM3 cells were seeded at a density of 5000 cells/well and treated with DMSO or 20 µM CBD for 24 h. Briefly, TM3 cells were fixed with 4% formaldehyde after treatment, permeabilized with 0.5% Triton™X-100 in 1 × PBS, blocked by 1 × Blocker™ BSA in PBS, and then stained with DyLight 488-Phalloidin (Catalog # 62249, ThermoFisher Scientific). Hoechst 33342 (Catalog # 62249, ThermoFisher Scientific) was used to stain cell nuclei. The resulting images were captured using a Cytation 5 Cell Imaging Reader (Agilent BioTek) with random fields selected. Samples obtained from three independent repeats were imaged and at least three images were captured for each sample.

### Cell cycle analysis

Cell cycle analysis was performed using propidium iodide (PI) staining to distinguish cell populations in G1, S, and G2/M phases based on their nucleic acid content as outlined in (Chen et al. 2020). TM3 cells were seeded at a density of 1.7 × 10^5^ cells/well in 6-well plates. After a 24 h treatment with 10–30 µM CBD, the cells were collected by trypsinization and centrifugation and cell numbers for each sample were adjusted to 1 × 10^6^ cells. Cells were resuspended in 300 µl of ice-cold 1 × PBS buffer, and then, 700 µl of ice-cold 70% ethanol was added to fix the cells for 1 h on ice. Subsequently, the TM3 cells were washed with 1 × PBS buffer and resuspended in 250 µL of 1 × PBS containing RNase A (200 µg/mL, Catalog # 19101, Qiagen, Valencia, CA) at 37 °C for 1 h to remove RNA. Then, 250 µL of 1 × PBS containing 10 µg/mL PI (Catalog # P1304MP, ThermoFisher Scientific) was added to each sample to stain the nucleic acids overnight at 4 °C. Flow cytometric analysis using a FACSCanto™ flow cytometer (BD Biosciences) was conducted to detect PI staining signals. Data were analyzed using FlowJo® software (Ashland, OR).

### Western Blot analysis

TM3 cells were seeded at a density of 1 × 10^6^ cells/dish in 100 mm dishes and then collected after being exposed to CBD at designated concentrations or DMSO for 24 h. Protein levels were analyzed by standard Western Blotting procedures using antibodies from Cell Signaling Technology (Danvers, MA) and Santa Cruz Biotechnology (Santa Cruz, CA): cyclin D1 (CST # 2978), cyclin D3 (CST # 2936), cyclin E2 (CST # 4132), CDK2 (CST # 2546), CDK4 (CST # 12790), CDK6 (CST # 3136), GAPDH (CST # 5174), anti-rabbit IgG-HRP (sc-2357), and anti-mouse IgG-HRP (sc-516102). Protein bands were captured by FluorChem E (ProteinSimple, San Jose, CA) and quantified by AlphaView software (San Jose, CA).

### 5-Ethynyl-2’-deoxyuridine (EdU) proliferation assay

DNA synthesis was measured by EdU incorporation using a Click-iT® EdU Flow Cytometry Assay kit (Catalog # C10425, ThermoFisher Scientific). TM3 cells were seeded at a density of 1.7 × 10^5^ cells/well in 6-well plates. After treatment with 0.1% DMSO, 20–30 µM CBD or 7-hydroxy-CBD, or 70–90 µM 7-carboxy-CBD for 24 h, the cells were collected by trypsinization and centrifugation and cell numbers for each sample were adjusted to 1 × 10^6^ cells. EdU staining and flow cytometric analysis were conducted according to the manufacturer’s instruction as described previously (Li et al. 2022). FACSCanto™ flow cytometer was used to detect cells that were actively dividing. Data were analyzed using FlowJo® software.

### Measurement of apoptosis using Annexin V-PI staining

Annexin V/PI staining was used to distinguish viable and apoptotic cells. Annexin V protein has a high affinity for phosphatidyl serine that is translocated to the outer leaflet of the plasma membrane in apoptotic cells. Therefore, cells stained positive for Annexin V are considered to be apoptotic cells. PI is impermeable in live cells but stains dead cells with red fluorescence. An Alexa Fluor® 488 Annexin V/Dead Cell Apoptosis Kit (Catalog # V13245, ThermoFisher Scientific) was used to differentiate live, early apoptotic, late apoptotic, and necrotic cells. TM3 cells were treated with the chemicals at designated concentrations and timepoints and collected for staining with Alexa Fluor® 488 Annexin V and 1 µg/mL PI for 15 min at room temperature in darkness. The samples were immediately analyzed using a FACSCanto™ flow cytometer with FACSDiva™ software and analyzed with FlowJo® software. The distribution of cells was plotted in two-dimensional dot plots.

### Measurement of population doubling levels

TM3 cells were incubated with either 0.1% DMSO or 20 µM or 30 µM CBD for 6 days. The population doubling (PD) was calculated as follows, PD = log (N2/N1)/log2, where N1 corresponds to the cell number at the initial time point, and N2 stands for the cell number at the subsequent time point. Specifically, 3.4 × 10^4^ of TM3 cells were seeded in 6-well plates for 24 h prior to treatment with CBD or DMSO. Day 0 was defined as the start of the chemical treatment. DMSO-treated TM3 cells reached 100% confluency on day 2 and therefore were split and counted every other day for a total of 6 days. At each split, the same number (3.4 × 10^4^) of DMSO-treated cells was re-seeded in a new culturing plate with fresh media-containing 0.1% DMSO. In a pilot study, we observed that the cell number of 30 µM CBD-treated cells did not exceed the initial seeded number (3.4 × 10^4^) on day 2 and, thus, reseeding could not be conducted. Therefore, the CBD-treated cells were kept in their initial plates, and media-containing 20 or 30 µM CBD was refreshed on days 2 and 4. The CBD-treated cells were counted on days 2, 4, and 6. Additionally, 30 µM CBD-treated TM3 cells detached on day 6, and thus, monitoring was discontinued on day 6.

### Quantification of CBD and its major metabolites by LC–MS/MS

The deuterated internal standards (CBD-D3, 7-hydroxy-CBD-D10, 7-carboxy-CBD-D10) were acquired from Cerilliant (Round Rock, TX). LC–MS grade formic acid, acetonitrile, and water were purchased from Fisher Scientific (Fair Lawn, NJ). Primary human Leydig and mouse TM3 cells were seeded at a density of 1.7 × 10^5^ cells/well in 6-well plates and treated with CBD, 7-hydroxy-CBD, and 7-carboxy-CBD for 24 h. At the end of the exposure, 1 mL of culture media was collected. The cells were then harvested by trypsinization and centrifugation. The number of viable cells in each sample was determined using trypan blue with a Countess 3 automated cell counter. The resulting cell pellet was resuspended in 200 μL of water and subjected to three freeze–thaw cycles. Afterward, the cellular suspension was centrifuged at 10,000 × g for 10 min at 4 °C, and the supernatant was collected and designated as the cell lysate. Twenty µL of either cell culture media or cell lysate was spiked with deuterium-labeled internal standards. Proteins were then precipitated using acetonitrile, and the supernatant was subsequently collected by centrifuging at 14,000 × g for 5 min at 4 °C.

The supernatant was introduced into an ACQUITY UPLC system paired with a Waters TQ-S mass spectrometer (Milford, MA). Analytes and their respective internal standards were eluted using a Waters ACQUITY UPLC HSS T3 column (2.1 mm × 100 mm × 1.8 μm) maintained at 45 °C. The mobile phase was composed of water (solvent A) and acetonitrile (solvent B), each containing 0.1% formic acid, with a flow rate of 0.4 mL/min. The elution started at 15% solvent B, which then transitioned with linear gradient from 15 to 100% solvent B over 8 min. This was held at 100% solvent B for 2 min, after which it decreased to 15% solvent B within 0.5 min and finally stabilized at 15% solvent B for an additional 1.5 min to allow for column re-equilibration. The eluted analytes were monitored using the TQ-S mass spectrometer equipped with an electrospray ion source operating in the positive-ion mode (ESI +) using multiple reaction monitoring (MRM). The monitored MRM transitions were m/z 315.2 > 193.0 for CBD, m/z 318.2 > 196.0 for CBD-D3, m/z 313.2 > 201.0 for 7-hydroxy-CBD, m/z 323.2 > 203.0 for 7-hydroxy-CBD-D10, m/z 345.2 > 299.2 for 7-carboxy-CBD, and m/z 355.2 > 309.2 for 7-carboxy-CBD-D10. Analytes were quantified using calibration curves that ranged from 0.5 to 2000 ng/mL with an r^2^ value exceeding 0.99. The quantification was performed with the Waters MassLynx 4.1 software (Milford, MA). Results were presented as ng of the analyte per 10^6^ cells.

### mRNA sequencing and data analysis

We used the same experimental settings that were used to monitor population doubling in the two types of Leydig cells over 5 days to extract RNA. 4.8 × 10^5^ cells were seeded in 150 mm dishes and allowed to attach for 24 h before the treatment. Day 0 marked the start of the treatment and cells were collected on day 1 to day 5. RNA samples were prepared and sequenced as previously described (Li et al. 2020). Briefly, total RNA from primary human Leydig and mouse TM3 cells was extracted using an RNeasy Mini kit (Qiagen, Valencia, CA). RNA samples were sent to Qiagen for mRNA sequencing, including library preparation using the QIAseq Stranded mRNA kit (Qiagen) and paired-ended sequencing using Illumina’s NovaSeq instrument. All reads have been mapped to the human genome (GRCh38) with ENSEMBL GRCh38.98 annotation or mouse genome Mus Musculus (GRCm38) with ENSEMBL GRCm38.98 annotation. Bioinformatics analyses including read mapping, quantification of gene expression, and analysis of differentially expressed genes (DEG) were carried out using Qiagen CLC Genomics Server 21.0.4. Specifically, "Empirical analysis of DEG" algorithm in CLC Genomics Workbench 21.0.4, utilizing the default settings, was used for differential expression analysis, which implements the “Exact Test” method for comparing two groups (Robinson and Smyth 2008; Robinson et al. 2010). The DEGs were defined as having a Bonferroni adjusted p value < 0.05 and absolute fold change ≥ 2. ~ 2500 to ~ 3300 DEGs from primary human Leydig cells and ~ 1200 to ~ 2300 DEGs from mouse TM3 cells were identified on day 1 to day 5 when comparing each treatment data with the control groups on the same day. Pathway enrichment methods were described previously (Liet al. 2018). Specifically, DEGs were analyzed using the Qiagen Ingenuity Pathway Analysis (IPA) or DAVID functional annotation tool (https://david.ncifcrf.gov/home.jsp) for pathway enrichment (Sherman et al. 2022). Gene ontology (GO) biological process database (GOTERM_BP_DIRECT) was used to annotate pathways. A biological process was considered significantly enriched when a Benjamini Hochberg adjusted *p* value was < 0.05. IPA comparison analyses were conducted to examine the time-course data and identify pathways that were changed with time. IPA also predicted the direction of pathway/upstream regulators changes, that is up-regulated, down-regulated, or affected determined by z-score. Z-score is a statistical measure to assess the significance of correlations between gene expression data and biological pathways or functions and is calculated by IPA through comparing the actual expression level to the expected pattern based on the literature. Z-score with absolute values equal to or larger than 2 was considered statistically significant, as defined in the User’s manual. In our presentation, blue colors represent down-regulation, and orange/red colors represent up-regulation.

### Statistical analysis

Data are presented as the mean ± standard deviation (SD) of three independent experiments. Statistical analyses were performed in GraphPad Prism 9 (San Diego, CA) using either one-way or two-way analysis of variance (ANOVA), followed by Dunnett’s Multiple comparisons test. Linear regression analyses were used to evaluate concentration-related trends. A significance level of *p* < 0.05 was considered statistically significant.

## Results

### Cytotoxic effects of CBD on TM3 mouse Leydig cells

**Fig. 1:**
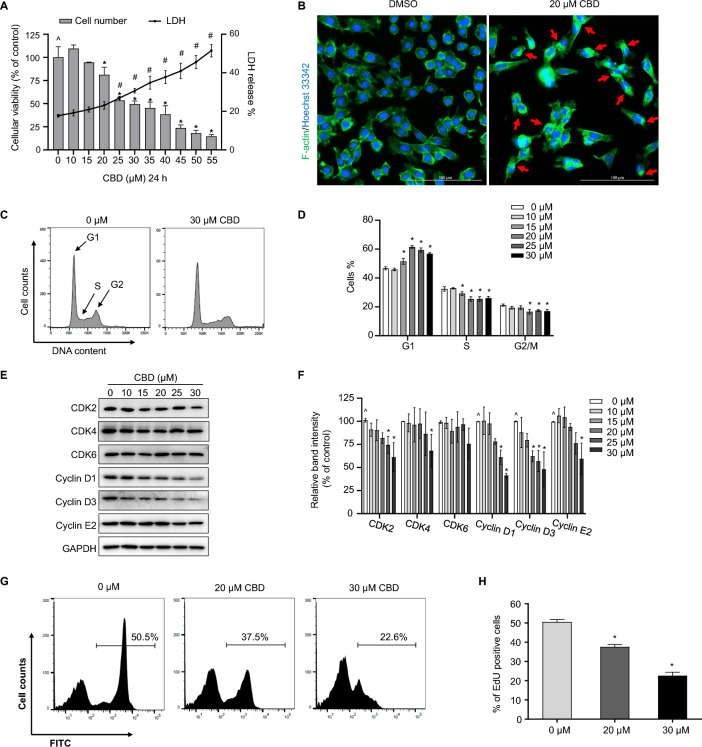
CBD is cytotoxic to TM3 mouse Leydig cells. **A** TM3 were treated with DMSO (0 μM) or CBD at concentrations of 10–55 µM for 24 h. Cytotoxicity was evaluated by counting viable cells and measuring the release of LDH. **B** Morphological changes induced by 20 µM CBD in TM3 cells. F-actin was stained with DyLight 488-phalloidin (green), and nuclei were counterstained with Hoechst 33342 (blue). Fluorescence microscopy images showed F-actin aggregation (indicated by red arrows). Scale bar (white) represents the length of 100 µm. Images were collected from three independent experiments and representative images are shown. **C** CBD arrests the cell cycle in TM3 cells at the G1 phase. The cell cycle distribution of TM3 cells was determined by measuring the content of propidium iodide-stained DNA using flow cytometry. **D** Bar graphs showing the mean DNA content with error bars representing standard deviations (SD, *n* = 3). **E** Total cellular proteins were extracted from TM3 cells treated with 10–30 µM CBD or DMSO for 24 h. Western blotting was performed to determine the levels of CDK2, CDK4, CDK6, Cyclin D1, Cyclin D3, and Cyclin E2. The intensity of each protein band was normalized to its corresponding GAPDH band. Representative images are shown in (**E**) and quantification is shown in (**F**). Bar graphs represent means ± SD (*n* = 3). **G** Representative histograms show the percentage of cells undergoing DNA synthesis (marked by a line). TM3 cells were treated with DMSO (0 µM), 20 µM, or 30 µM CBD for 24 h before analyzing DNA synthesis ability. **H** Quantification of results from (**G**); the bar graph shows the average percentage of EdU-positive cells ± SD (*n* = 3). ^^^, significant concentration-dependent linear trend. *, *p* < 0.05, significantly different from the control group under the same treatments

We first investigated the potential cytotoxic effects of CBD on TM3 cells. As shown in Fig. 1A, we observed a concentration-dependent decrease in cell viability of CBD-treated groups at 24 h, in comparison to the control group. The BMD_50_ of CBD was 29.7 µM (Table 1). Additionally, an elevation in LDH release was observed as the concentration of CBD treatment increased (Fig. 1A). Specifically, starting at 25 µM, CBD caused significant intracellular LDH release, indicating that cell damage occurred upon CBD treatment. We also characterized the morphological changes of CBD-treated TM3 cells by staining the filamentous actin (F-actin), a major cytoskeleton component that is responsible for maintenance of cell shape, cell movement, and division (Gibieza and Petrikaite 2021). As shown in Fig. 1B F-actin aggregated (indicated by the red arrows) in TM3 cells treated with a sub-lethal (20 µM) concentration of CBD after 24 h, as compared to the control cells. The aggregation of F-actin may contribute to CBD-induced cellular toxicity, such as failure in cell division (Castro et al. 2012).

**Table 1:** The BMD_50_ of CBD, 7-hydroxy-CBD, and 7-carboxy-CBD in primary human Leydig and TM3 cells

Cell type	Primary human Leydig cells BMD_50_^&^	TM3 BMD_50_
CBD	21.6 (20.0–22.8)^#^	29.7 (26.3–32.9)
7-Hydroxy-CBD	30.2 (29.1–31.9)^*^	35.3 (23.2–39.4)
7-Carboxy-CBD	127.3 (113.6–141.8)^#, *^	95.8 (86.4–105.9)^^^

^*^significantly different from CBD treatment in primary human Leydig cells. ^^^, significantly different from CBD treatment in TM3 cells

^#^significantly different from TM3 cells treated with the same chemical

Primary human Leydig cells and TM3 cells were incubated with designated concentrations of CBD, 7-hydroxy-CBD, or 7-carboxy-CBD for 24 h. The half inhibitory concentrations were calculated by Bayesian benchmark dose and the data were presented as the BMD_50_ (BMDL, 5th percentile–BMDU,95th percentile). ^&^, data published in (Li et al. 2023a)

We previously reported that CBD can arrest cell cycle progression in primary human Sertoli and Leydig cells in vitro, two major male reproductive cell types within testes (Li et al. 2023a, b; Li et al. 2022). To examine if the similar mechanism applies to TM3 cells, we analyzed the cell cycle by staining the nucleus with PI at concentrations ≤ BMD_50_ (30 µM). As shown in Fig. 1C and D, treatment with 30 µM CBD led to an increase of G1 phase portion from 46.7% to 56.8% with a concurrent decrease of S-phase percentage from 32.4% to 26.1% and a decrease of G2/M phase percentage from 21.2% to 17.0%, compared to the control group. Therefore, 15—30 µM CBD induced G1 arrest in TM3 cells, leading to a decrease in cell number because of the stalled cell cycle.

We subsequently explored the mechanism underlying cell cycle arrest by analyzing the protein levels of cell cycle regulators, including kinases CDK2, CDK4, CDK6, and cyclins D1, D3, and E2. There was a concentration-dependent decrease of CDK2 and cyclins D1, D3, and E2 in TM3 cells treated with 10–30 µM CBD (Fig. 1E and F). In addition, CDK4 decreased by 31.8% at 30 µM CBD while remaining unchanged at concentrations lower than 30 µM (Fig. 1E and F). The protein level of CDK6 was not changed by 10–30 µM CBD (Fig. 1E and F). Previously, it was reported that down-regulation of CDK2 in human embryonic stem cells can cause G1 arrest (Neganova et al. 2009). The reduction of protein levels of CDKs and cyclins supported the observed phenotype of cell cycle arrest at the G1 phase, allowing the cell to check for any cellular damage before proceeding to S phase.

We used EdU staining to monitor DNA synthesis activities and track the number of cells in the S phase. Treatment with 20 and 30 µM of CBD treatment for 24 h reduced the percentage of S-phase cells by 13.0% and 27.9%, respectively, (Fig. 1G and H), which confirms our previous observation (Fig. 1D) that CBD treatment reduces the number of cells in the S phase.

### Cytotoxic effects of 7-carboxy-CBD and 7-hydroxy-CBD on TM3 cells

**Fig. 2:**
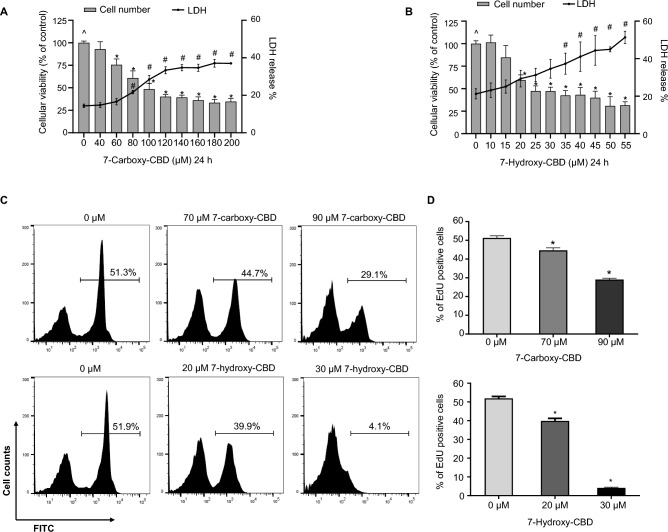
7-Carboxy-CBD and 7-hydroxy-CBD are cytotoxic to TM3 mouse Leydig cells. **A** and **B** TM3 cells were exposed to 40–200 µM 7-carboxy-CBD or 10–55 µM 7-hydroxy-CBD for 24 h. Live cell numbers and the release of LDH were measured. **C** Inhibition of DNA synthesis in TM3 cells by 7-carboxy-CBD and 7-hydroxy-CBD. TM3 cells were treated with DMSO (0 μM), 70 or 90 µM 7-carboxy-CBD, or 20 or 30 µM 7-hydroxy-CBD for 24 h. Representative histograms show the percentage of EdU incorporation in TM3 cells. **D** Bar graphs represent the average percentage of EdU-positive cells ± SD (*n* = 3). ^^^, significant concentration-dependent linear trend. *, *p* < 0.05 and significantly different from the DMSO control

We next sought to determine the cytotoxic effects of CBD’s main metabolites, 7-carboxy-CBD and 7-hydroxy-CBD, on TM3 cells. The two metabolites reduced cell viability in a concentration-dependent manner (Fig. 2A and B). After 24 h treatment, the BMD_50_ of 7-carboxy-CBD and 7-hydroxy-CBD were 95.8 µM and 35.3 µM, respectively (Table 1). 7-Hydroxy-CBD had a comparable BMD_50_ with CBD (29.7 µM), whereas 7-carboxy-CBD was the least toxic in TM3 cells (Table 1). LDH release was increased as the concentration of 7-carboxy-CBD and 7-hydroxy-CBD increased, which indicates that cell damage also occurred as the cell viability decreased. To explore whether the two CBD metabolites inhibited DNA synthesis, EdU labeling was used to quantify the changes in S-phase cells. As shown in Fig. 2C and D, TM3 cells treated with 70 or 90 µM of 7-carboxy-CBD exhibited a decreased percentage of EdU-positive cells to 44.7% and 29.1%, respectively, compared to 51.3% in the control group. Similarly, TM3 cells treated with 20 or 30 µM of 7-hydroxy-CBD showed a decreased percentage of EdU-positive cells to 39.9% and 4.1%, respectively, compared to 51.9% in the control group. Therefore, 7-carboxy-CBD and 7-hydroxy-CBD exhibit the similar profiles in inhibiting DNA synthesis as the parental drug CBD in TM3 cells.

### Induction of apoptosis in TM3 cells by CBD and its main metabolites, 7-hydroxy-CBD, and 7-carboxy-CBD

**Fig. 3:**
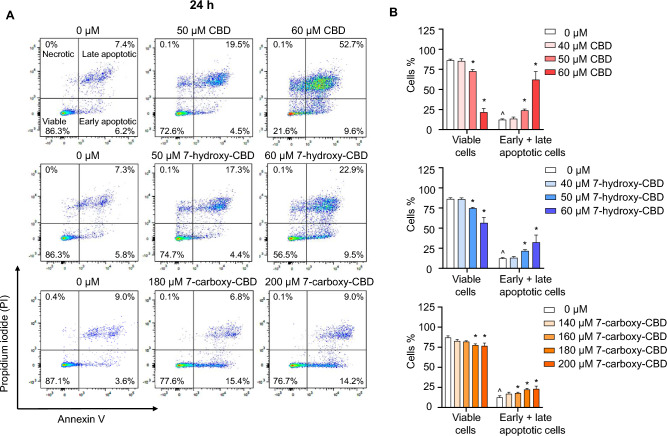
Induction of apoptosis by CBD, 7-hydroxy-CBD, and 7-carboxy-CBD in TM3 cells. TM3 cells were exposed to DMSO (0 µM), 40–60 µM CBD, 40–60 µM 7-hydroxy-CBD, or 140–200 µM 7-carboxy-CBD for 24 h. The increase in the percentage of cells stained positive for Annexin V was measured as an indicator of apoptosis. **A** Representative scatter plots display the distribution of cells based on Annexin V (X-axis) and PI (Y-axis) staining. **B** Quantitation of results from (**A**); the bar graph shows the mean percentage of viable and early and late apoptotic cells ± SD (*n* = 3). ^^^, significant concentration-dependent linear trend. *, *p* < 0.05 and significantly different from the control group

We next conducted Annexin V/PI staining to monitor the occurrence of apoptosis. After a 24 h treatment, we observed that 50—60 µM CBD, 50—60 µM 7-hydroxy-CBD, and 160—200 µM 7-carboxy-CBD treatments led to a concentration-dependent increase in the percentage of cells undergoing early and late apoptosis, as indicated by positive Annexin V-staining (Fig. 3). Specifically, 60 µM of CBD, 60 µM of 7-hydroxy-CBD, and 200 µM of 7-carboxy-CBD increased the percentage of early and late apoptotic cells by 49.9%, 20%, and 10.7%, respectively, compared to the control group.

**Fig. 4:**
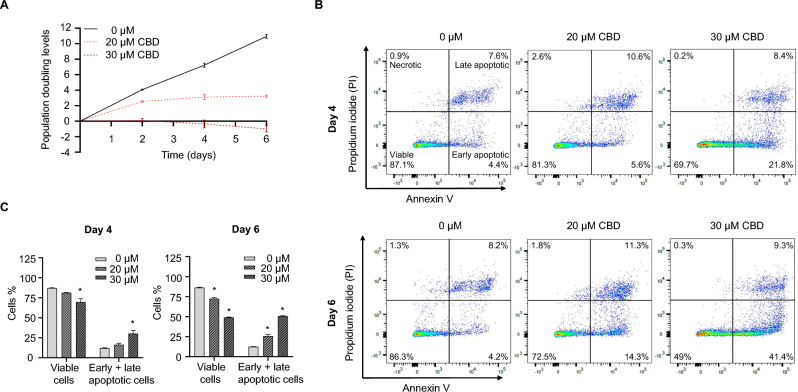
CBD induced apoptosis at sub-lethal concentrations during 6 days of incubation in TM3 cells. **A** Growth curves of TM3 cells treated with DMSO, 20, or 30 µM CBD for 6 days. **B** Detection of apoptotic TM3 cells by Annexin V/PI staining on day 4 or 6. TM3 cells were treated with DMSO (0 μM), 20 µM CBD, or 30 µM CBD for 4 or 6 days. Representative scatter plots show the results of Annexin V (X-axis)/ PI (Y-axis) staining. **C** Quantification of results from (**B**). Bar graphs represent the average percentage of viable and early and late apoptotic cells from three independent experiments. *, *p* < 0.05 and significantly different from the DMSO control

We further assessed the long-term effects of CBD at concentrations lower than BMD_50_ by conducting a 6-day growth monitoring of TM3 cells treated with CBD. The population doubling level (PDL) shows the total number of times the cell population has doubled at specific time points. We observed a steady increase in the growth of DMSO- (0 μM) treated TM3 cells, with the PDL increasing by ~ 3.6 at each timepoint (Fig. 4A). In contrast, growth inhibition and cell number decrease were observed in the 30 µM CBD-treated group, particularly on days 4 and 6 (Fig. 4A). Simultaneously, we monitored time-dependent apoptosis induced by 20 and 30 µM CBD using Annexin V/PI staining. We observed that 30 µM CBD induced early and late apoptosis in TM3 cells on days 4 and 6 (Fig. 4B and C). The flow cytometric analysis of 30 µM CBD-treated TM3 cells showed a 17.4% and 37.3% decrease of viable cells and a 18.2% and 38.3% increase of early and late apoptotic cells on days 4 and 6 (Fig. 4B and C). Furthermore, the percentage of early and late apoptotic cells increased as the concentration of CBD increased from 20 µM to 30 µM and as time proceeded (Fig. 4B and C), confirming that CBD-induced apoptosis in TM3 cells is concentration- and time-dependent.

### Comparison of cellular uptake between TM3 and primary human Leydig cells upon treatment by CBD, 7-hydroxy-CBD, and 7-carboxy-CBD

As shown in Table 1, the BMD_50_ analysis comparison indicated that CBD was more cytotoxic in primary human Leydig cells compared to TM3 cells. The BMD_50_ of 7-hydroxy-CBD was not significantly different between two types of cells, while 7-carboxy-CBD was slightly more cytotoxic to TM3 compared to primary human Leydig cells.

To understand the toxicity differences of CBD, 7-hydroxy-CBD, and 7-carboxy-CBD in human and mouse Leydig cells, we studied the cellular uptake of these chemicals. Different cellular uptake capacity of a cell type could contribute to different cytotoxic effect of a chemical. Cells that have higher uptake capacity for a toxic chemical are more likely to accumulate higher concentrations of the chemical in their intracellular compartments, potentially leading to greater cytotoxicity.

**Fig. 5:**
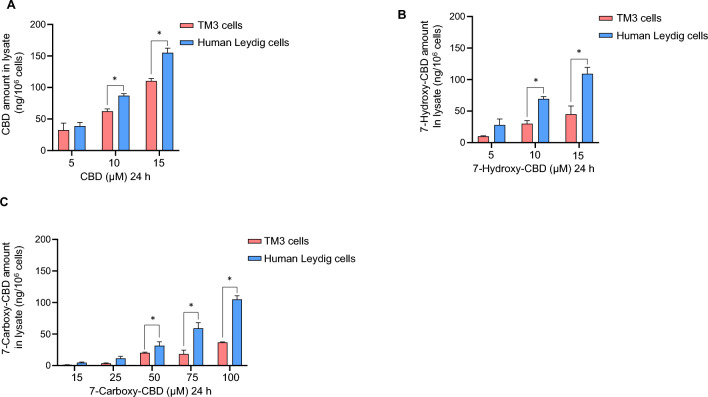
Mass spectrometry analysis of CBD, 7-hydroxy-CBD, and 7-carboxy-CBD in primary human Leydig and TM3 cells. Primary human Leydig and TM3 cells were exposed to 5—15 µM CBD (**A**), 5—15 µM 7-hydroxy-CBD (**B**), or 15—100 µM 7-carboxy-CBD (**C**) for 24 h. One mL of media supernatants and cell pellets were collected. The normalized amount of CBD, 7-hydroxy-CBD, and 7-carboxy-CBD in the cell lysate of primary human Leydig and mouse TM3 cells is shown in (**A**–**C**). The bar graphs show the mean ± SD from three independent experiments. *, p < 0.05, indicating a significant difference between primary human Leydig cells and TM3 cells

Our mass spectrometry analyses indicated that CBD, 7-hydroxy-CBD, and 7-carboxy-CBD were present in a concentration-dependent manner in the cell lysates of both cell types (Fig. 5A–C). We detected higher levels of CBD, 7-hydroxy-CBD, and 7-carboxy-CBD in cell lysates of primary human Leydig cells, in comparison to TM3 cells exposed to the same concentrations (Fig. 5A–C). When comparing the intracellular amount of the three chemicals after exposure at the same concentration of 15 µM, the average quantities in cell lysates of TM3 were as follows: CBD (110.4 ng/10^6^ cells) > 7-hydroxy-CBD (45.0 ng/10^6^ cells) > 7-carboxy-CBD (1.3 ng/10^6^ cells). Similarly, in primary human Leydig cells, the average quantities were CBD (155.0 ng/10^6^ cells) > 7-hydroxy-CBD (109.3 ng/10^6^ cells) > 7-carboxy-CBD (4.7 ng/10^6^ cells). 7-Carboxy-CBD’s uptake was about 100-fold less than that of CBD in both types of Leydig cells. This may explain why cytotoxicity of 7-carboxy-CBD was the lowest among three chemicals in both types of Leydig cells.

### Transcriptomic analysis of time-course CBD treatment in primary human Leydig and TM3 cells

**Fig. 6:**
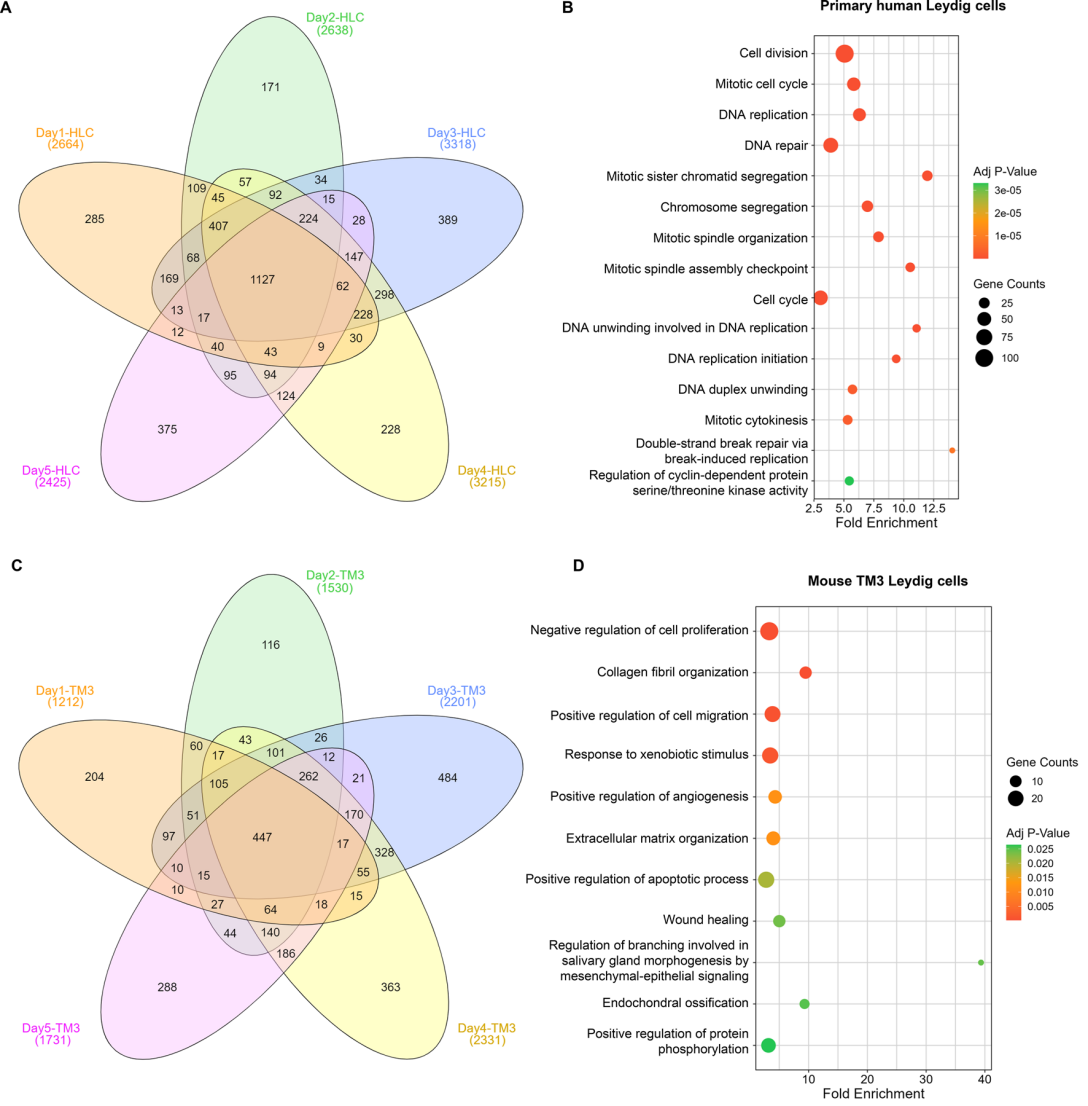
Transcriptomic analysis of CBD-treated primary human Leydig and TM3 cells for 5 days. **A** and **C** Venn diagrams showing the number of differentially expressed genes on day 1, 2, 3, 4, and 5 from primary human Leydig cells (**A**) and TM3 cells (**C**). Numbers on each section represent the numbers of differentially expression genes. Venn diagrams were created using the online tool: http://www.interactivenn.net/. The dark orange-colored center area in the Venn diagrams denotes the number of overlapped differentially expressed genes during days 1 to 5. **B** and **D** Bubble plots illustrating enriched biological processes (GOTERM_BP_DIRECT) using overlapped differentially expressed genes from day 1 to 5 in primary human Leydig cells (**B**) and TM3 cells (**D**). Adjusted p values were calculated using a Benjamini–Hochberg correction

To investigate the molecular changes induced by CBD and the progression of apoptosis in Leydig cells, we conducted a transcriptome analysis to examine alterations on mRNA levels. RNA samples were collected from primary human Leydig cells treated with 15 µM CBD and TM3 cells treated with 30 µM CBD and their respective DMSO controls each day for 5 consecutive days. We selected these treatment conditions, because CBD at these concentrations for 5 days caused significant cellular growth inhibition and cell death in primary human Leydig cells (Li et al. 2023a) and TM3 cells (Fig. 4). Pairwise comparisons were performed between the control group and the CBD-treatment groups on each day. As shown in Fig. 6A, we obtained ~ 2400 to ~ 3300 DEGs in primary human Leydig cells samples based on an absolute fold change ≥ 2 and adjusted p value ≤ 0.05 for each time point. Similarly, ~ 1200 to ~ 2300 DEGs were found in TM3 cells (Fig. 6C). As shown in Venn diagrams (Fig. 6A and B), there were 1127 genes identified in primary human Leydig cells and 447 genes identified in TM3 cells that were commonly altered at all time points. These overlapped genes were used to conduct pathway enrichment analysis, as depicted in bubble graphs illustrating the top 15 GO biological processes/pathways. In primary human Leydig cells, cell division, mitotic cell cycle, and DNA replication were ranked highest (Fig. 6B), a result consistent with our previous observation in which CBD disturbed the cell cycle and inhibited DNA replication in human Leydig cells at 24 h (Li et al. 2023a). In TM3 cells, pathways, such as negative regulation of cell proliferation, extracellular matrix organization, and positive regulation of protein phosphorylation, were enriched (Fig. 6D). In addition, positive regulation of the apoptotic process was enriched in TM3 cells, which supports our previous observation that CBD induced apoptosis in TM3 cells (Figs. 3 and 4).

### mRNA sequencing revealed CBD induced stress-related cellular responses in primary human Leydig and TM3 cells

**Fig. 7:**
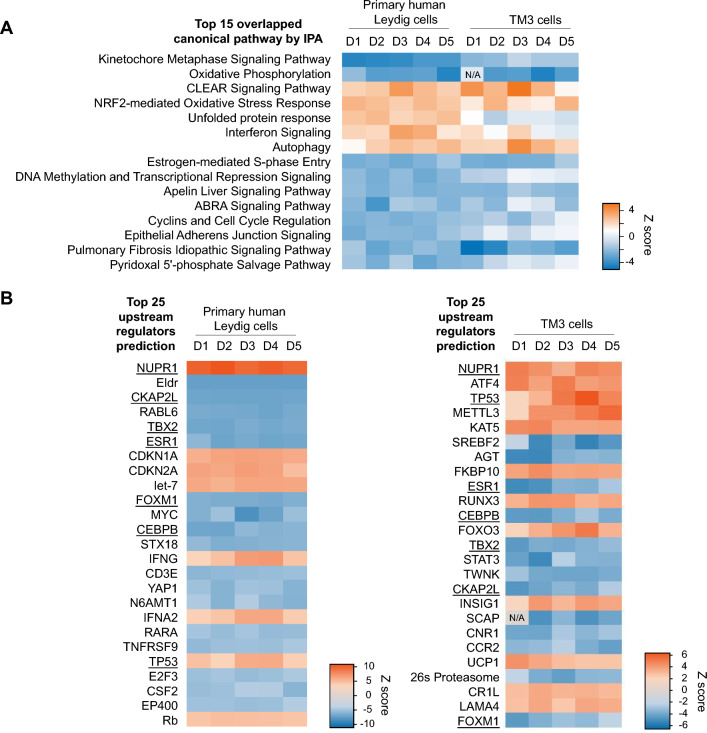
Enrichment of canonical pathway and upstream regulators prediction in CBD-treated primary human Leydig and TM3 cells using Ingenuity Pathway Analysis. **A** Heatmap visualization of top canonical pathways that overlap in primary human Leydig and TM3 cells using genes differentially expressed on each day comparing negative control and CBD-treated cells from day 1 to 5. **B** Heatmap visualization of top predicted upstream regulators from the differentially expressed genes from days 1 to 5. Overlapped upstream regulators are underlined. Blue represents down-regulation and orange represents up-regulation upon CBD treatment

Time-course analysis using data from day 1 to day 5 was performed using IPA-based comparison analysis, and we summarized the top 15 overlapped canonical pathways enriched from primary human Leydig and TM3 cells (ranked according to the results of primary human Leydig cells). As shown in Fig. 7A, most overlapped pathways show a similar trend of activation or inhibition in response to CBD treatments in both types of Leydig cells. For example, the coordinated lysosomal expression and regulation (CLEAR) signaling pathway that responds to cellular stress was up-regulated in both primary human Leydig and TM3 cells. Similarly, the NRF2-mediated oxidative stress response and autophagy (“self-digestion”) show up-regulation in both types of Leydig cells. Conversely, pathways related to mitosis, such as kinetochore metaphase signaling pathway and cyclins and cell cycle regulation were down-regulated. Moreover, oxidative phosphorylation was down-regulated in both cell types; down-regulation of this pathway is related to mitochondrial dysfunction. As a result, these altered pathways can be upstream events and contribute to the occurrence of apoptosis in primary human Leydig and TM3 cells. Interestingly, two pathways, unfolded protein response and interferon signaling, changed differently in the two cell types. The unfolded protein response pathway is recognized as a key regulator in response to endoplasmic reticulum (ER) stress (Read and Schroder 2021), while the interferon signaling pathway exhibits antiviral and growth inhibitory effects (Platanias 2005). These two pathways were up-regulated in primary human Leydig cells at all time points, while they were down-regulated in TM3 cells.

Besides similar pathway enrichment results, the predicted up-regulated upstream regulators in primary human Leydig cells and TM3 also had overlaps, such as NUPR1 (Nuclear Protein 1) and TP53 (Tumor Protein P53) (underlined in Fig. 7B). In particular, the z-scores (as a measure of significance) of TP53 in both primary human Leydig and TM3 cells increased as time proceeded from day 1 to day 4 (Fig. 7B). This prediction is consistent with our previous finding that p53 was involved in the cytotoxicity induced by CBD in human Sertoli cells, another essential cell type in testes (Li et al. 2023b). In addition, NUPR1 appeared to be a strong activated upstream regulator in primary human Leydig cells. NUPR1 has been reported to form a complex with p53 and regulate genes involved in cell cycle progression and apoptosis (Clark et al. 2008).

### Gene expression changes supported the induction of apoptosis by CBD in primary human Leydig and TM3 cells

**Fig. 8:**
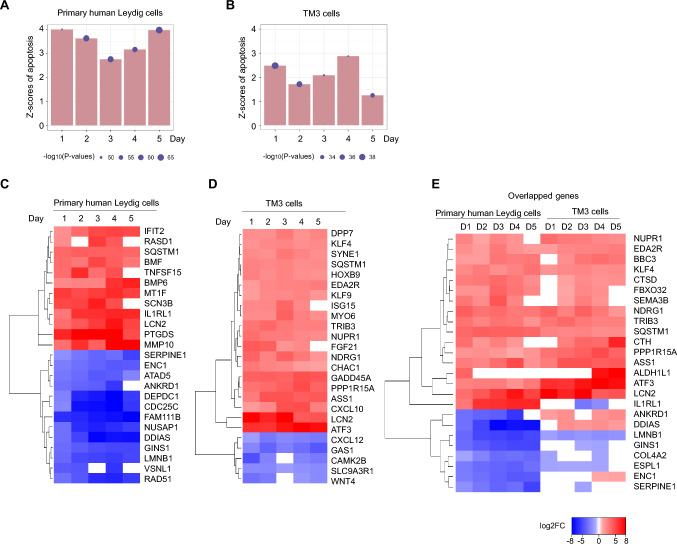
Transcriptomic analysis revealed the up-regulation of apoptosis in CBD-treated primary human Leydig and TM3 cells. **A** and **B** Bar graphs represent the z-score of apoptosis (generated by Ingenuity Pathway Analysis) in two types of Leydig cells. Red bar represents the prediction of up-regulated apoptosis. A higher positive z-score suggests a stronger correlation between the up-regulation of apoptosis-related genes and the predicted activation of apoptosis. Purple dot indicates the corresponding p value. For a *p*-value of 0.05, the −log_10_(0.05) value is 1.3. Larger dots correspond to smaller p values. **C**–**E** Heatmaps with hierarchical clustering showing expression levels of differentially expressed genes supporting the occurrence of apoptosis in human Leydig and mouse TM3 cells. Gene expression level was plotted by log_2_ (fold change). Blue colors represent down-regulation of gene expression and red colors represent up-regulation

The results of mRNA sequencing supported the occurrence of apoptosis in both primary human Leydig and TM3 cells upon CBD treatments. In primary human Leydig cells, the z-scores of apoptosis for all 5 days observed were larger than 2 (Fig. 8A). A positive z-score indicates the prediction of the up-regulation of apoptosis and an absolute z-score of apoptosis ≥ 2 is considered significant (Qiagen Ingenuity Downstream Effects Analysis in IPA 2024). Similarly, in TM3, the z-score of apoptosis was larger than 2 on days 1, 3, and 4 (Fig. 8B). To understand the molecular changes during apoptosis, we focused on genes whose expression changes affect the apoptosis pathway, as determined by IPA. Figure 8C and D show representative apoptosis-related genes with maximum absolute fold change at each time points in primary human Leydig and TM3 cells, respectively. Figure 8E summarizes the overlapped genes that positively regulate apoptosis in both types of cells. Most genes were regulated in a similar manner in both primary human Leydig and TM3 cells. Among these genes, the BBC3 (BCL2 binding component 3, commonly known as PUMA) gene encodes a pro-apoptotic Bcl-2 family member that is induced by p53 and can activate Bax and/or Bak proteins to pierce the mitochondrial outer membrane and promote apoptosis (Hemmati et al. 2010; Villunger et al. 2003). The ATF3 gene (activating transcription factor 3) encodes a member of the ATF/CREB (cAMP-responsive element-binding protein) family of transcription factors that is often induced in response to cellular stress, such as DNA damage or ER stress, which further contributes to apoptosis (Ku and Cheng 2020; Zhao et al. 2016).

## Discussion

This study examined the in vitro effects of CBD and its two major metabolites, 7-carboxy-CBD and 7-hydroxy-CBD, on TM3 mouse Leydig cells and compared the molecular mechanisms of CBD-induced cytotoxicity between TM3 cells and previously reported findings in primary human Leydig cells. We demonstrated that CBD treatment induced cytotoxicity, disturbed the cell cycle, inhibited DNA synthesis, and induced apoptosis in TM3 mouse Leydig cells (Figs. 1 and 3). These outcomes are similar between human and mouse Leydig cells. Additionally, the cellular uptakes of CBD, 7-hydroxy-CBD, and 7-carboxy-CBD were compared between human and mouse Leydig cells. Interestingly, a lesser amount (Fig. 5C) but higher toxicity of 7-carboxy-CBD (Table 1) was found in TM3 cells than primary human Leydig cells. These results indicate that TM3 cells may be more vulnerable to 7-carboxy-CBD-induced cytotoxicity. Previously, it had been demonstrated that CBD can be metabolized to 7-hydroxy-CBD and further converted to 7-carboxy-CBD by cytochrome P450 enzymes (CYP), such as CYP2C9, 2C19, and 3A4 (Beers et al. 2021; Nasrin et al. 2023). Additionally, a minor metabolic pathway involves the conjugation of 7-carboxy-CBD to form 7-carboxy-CBD-glucuronide by UDP glucuronosyltransferase (UGT), such as UGT1A9 and 2B7 (Nasrin et al. 2023). The relatively lower cellular amount of 7-carboxy-CBD compared to CBD and 7-hydroxy-CBD may be attributed to its potentially limited cellular uptake, possibly influenced by its larger size, more polar carboxy group, reduced lipophilicity, further metabolism by UGT enzymes, and involvement of unidentified transporters. Moreover, whether differences in membrane composition, transporters expression levels, and membrane surface area contribute to the different cellular uptake of 7-carboxy-CBD between mouse and human Leydig cells warrants further investigation.

Our mRNA-sequencing analyses confirmed the involvement of cell cycle/cell proliferation and apoptosis pathways in CBD-induced cytotoxicity in primary human Leydig cells and mouse TM3 cells. These findings align with previous studies’ observations that CBD induced apoptosis in various cell types, including human leukemia, glioma, and gastric cancer cells (Jeong et al. 2019; Massi et al. 2006; Mckallip et al. 2006). Moreover, the pro-apoptotic property of CBD in normal lymphocytes has been reported to cause immunosuppressive effects (Kozela et al. 2010). Our observation that CBD-induced apoptosis in human and mouse Leydig cells indicate a potential mechanism underlying the male reproductive toxicity of CBD in testes. Considering that adult Leydig cells lack the ability to self-renew, their apoptotic cell death can lead to decreased number of Leydig cells, which are responsible for producing testosterone. In monkeys and mice, CBD treatment has been reported to decrease testosterone levels (Carvalho et al. 2018; Rosenkrantz and Esber 1980). The underlying mechanism for the decreased testosterone remains to be further determined; however, CBD-induced apoptotic death of Leydig cells could be a contributing factor.

Our transcriptomic analyses in human and mouse Leydig cells demonstrated that CBD treatment caused the up-regulation of several pathways that respond to cellular stress, such as lysosome and mitochondrial dysfunction, oxidative stress, autophagy, and the upstream regulator p53. In response to these stresses, cells may temporarily pause the cell cycle to cope with adverse conditions. However, when cellular stress becomes overwhelming, apoptosis becomes the choice for eliminating dysfunctional cells. Meanwhile, the activation of p53 can also lead to cell cycle arrest to stop the proliferation of damaged cells (Ozaki and Nakagawara 2011). Therefore, these signaling pathway changes may serve as underlying mechanisms for CBD’s toxicity, triggering apoptosis in primary human Leydig and TM3 cells. These possible mechanisms are supported further by previous research on CBD in different cell types. For example, CBD increased the level of cellular reactive oxygen species and induced apoptosis in murine thymocytes, EL-4 thymoma cells, and human glioma cells (Lee et al. 2008; Massi et al. 2006). CBD inhibited proliferation, impaired mitochondrial activity, and induced apoptosis in human and canine glioma cells (Gross et al. 2021). CBD directly targets mitochondria, disturbs calcium homeostasis, and induces autophagy in acute lymphoblastic leukemia (Olivas-Aguirre et al. 2019).

One of the main functions of Leydig cells is to synthesize and secrete testosterone. However, TM3 cell line may not be a suitable model to test such a function because we failed to detect a measurable amount of secreted testosterone using a homogeneous time-resolved fluorescence assay (testosterone HTFR kit, Cisbio, detection limit: 0.1 nM) or enzyme-linked immunosorbent assay (testosterone ELISA kit, Enzo Life Sciences, sensitivity: 5.67 pg/mL). We also conducted RT-PCR assays to examine the expression of key steroidogenic genes in TM3 cells to identify any changes in the testosterone biosynthesis pathway (data not shown). The genes *Lhcgr* (luteinizing hormone/choriogonadotropin receptor), *Cyp11a1* (cytochrome P450 family 11 subfamily A member 1), *Hsd3b1* (3β-hydroxysteroid dehydrogenase 1), *Cyp17a1* (cytochrome P450 family 17 subfamily A member 1), and *Hsd17b3* (17β-hydroxysteroid dehydrogenase 3) were not expressed in TM3 cells (Ct values were above 35 and considered as non-detectable). The gene *Star* (steroidogenic acute regulatory protein), a key gene that regulates cholesterol transfer, was slightly expressed with a Ct value around 30 but the StAR protein was not detectable in Western Blot (data not shown). By contrast, using the mouse testis tissue as a positive control, the Ct values of *Lhcgr*, *Cyp11a1*, *Hsd3b1*, *Cyp17a1*, *Hsd17b3,* and *Star* were similar to *Gapdh* (the reference gene), suggesting the abundance of these transcripts in vivo. StAR protein from the mouse testis tissue sample was also detected with Western blot (data not shown). Therefore, the absent expression of *Lhcgr*, *Cyp11a1*, *Hsd3b1*, *Cyp17a1*, *Hsd17b3,* and *Star* confirmed that the testosterone synthesis pathway was not functional, which aligns with our findings that testosterone secretion was scarcely detectable in the present in vitro setting. In good agreement with our results, the TM3 cell line was recently reported to be unsuitable for studying gonadal androgen biosynthesis, because it lacks the last critical steroidogenic enzyme, 17β-hydroxysteroid dehydrogenase 3 (17β-hsd3), to synthesize testosterone (Engeli et al. 2018). In summary, the TM3 cell line may not be a great model for investigating testosterone production and studying the regulation of the testosterone synthesis pathway in vitro, as this pathway is non-functional in TM3 cells. Despite the deficiency in testosterone production, it is important to note that TM3 cells, being a Leydig cell line, still exhibit sensitivity to CBD-induced toxicity. This observation aligns with earlier reports of CBD-induced Leydig cell toxicity.

In this study, we demonstrated that CBD and its major two metabolites were cytotoxic to TM3 mouse Leydig cells, and the cell responses were similar between mouse Leydig cells and previously reported findings in human Leydig cells. Using transcriptomic analyses, we further demonstrated that primary human Leydig cells and mouse Leydig cells shared some molecular mechanisms underlying CBD’s cytotoxicity, while differences also existed. These findings shed light on the potential hazards associated with CBD usage, particularly concerning male reproductive toxicity, and provide insights into the molecular mechanisms involved in CBD-induced cytotoxicity in Leydig cells.

## Data Availability

Data available on request from the authors.
